# Remission Induced by Shichimotsukokato in an Older Adult With Nephrotic Syndrome Secondary to Diabetic Kidney Disease: A Case Report

**DOI:** 10.7759/cureus.81115

**Published:** 2025-03-24

**Authors:** Kazushi Uneda, Tatsuro Kikuchi, Masayuki Mori, Akira Kaneko, Eiichi Tahara

**Affiliations:** 1 Department of Kampo Medicine, Aizu Medical Center, Fukushima Medical University, Aizuwakamatsu, JPN

**Keywords:** complementary and integrative medicine, diabetic kidney disease, kampo formula, kampo medicine, nephrotic syndrome, shichimotsukokato

## Abstract

Nephrotic syndrome is a major renal disease characterized by massive proteinuria, hypoalbuminemia, systemic edema, and hyperlipidemia. Diabetic kidney disease (DKD) can induce secondary nephrotic syndrome, which is often challenging to manage with conventional treatments such as renin-angiotensin system inhibitors and diuretics. We present a case of a 90-year-old woman with DKD who developed nephrotic syndrome secondary to DKD that was refractory to standard treatments. Upon administration of shichimotsukokato (SCMKT), a representative Kampo formula created in Japan, there was a marked reduction in the proteinuria and an improvement in the serum albumin concentration, leading to remission of the nephrotic syndrome and enabling tapering of the renin-angiotensin system inhibitor and diuretics. This case suggests that SCMKT may be a candidate therapeutic option for nephrotic syndrome in patients with DKD. Further studies are needed to clarify the clinical benefits and pharmacological mechanisms of SCMKT in DKD.

## Introduction

Nephrotic syndrome is a major renal disease characterized by proteinuria, hypoalbuminemia, systemic edema, and hyperlipidemia [1]. One of the frequent causes of secondary nephrotic syndrome in adult patients is diabetic kidney disease (DKD) [2,3]. Although nephrotic syndrome secondary to DKD is managed with conventional treatments, such as renin-angiotensin system inhibitors and diuretics [1], and new therapies, such as sodium-glucose cotransporter 2 inhibitors and nonsteroidal mineralocorticoid receptor antagonists [4], some cases are challenging to treat. Therefore, new therapeutic approaches are needed.

Kampo medicine is a Japanese traditional therapy that includes herbal treatments [5]. Because 148 ethical Kampo formulas are covered by the Japanese health insurance system as well as modern Western medical practices, over 80% of Japanese physicians prescribe Kampo formulas in their daily clinical situations [6]. Kampo medicine has historically been used to treat renal diseases, including nephrotic syndrome [7]. Shichimotsukokato (SCMKT) is a Kampo formula created in Japan (http://mpdb.nibiohn.go.jp/stork/). Based on the concept of Kampo medicine, SCMKT typically targets the pathophysiology in older hypertensive patients with blood deficiency patterns (nutritional and functional disorders of the skin and organs). Therefore, SCMKT is a potential candidate for treating renal diseases, hypertension, and atherosclerosis in older patients. Previous basic studies have demonstrated the renoprotective and antihypertensive effects of SCMKT [8,9]. However, clinical reports of the use of SCMKT in patients with DKD are limited.

In this case report, we describe an older adult patient with secondary nephrotic syndrome due to DKD who showed substantial improvements after the administration of SCMKT.

This study was partially presented at the 40th Annual Meeting of The Japan Society for Oriental Medicine (JSOM) Tohoku.

## Case presentation

A 90-year-old woman with limb edema presented to our hospital. She had a 14-year history of type 2 diabetes mellitus and hypertension. After a detailed examination conducted by a nephrologist, she was diagnosed with DKD. Despite dietary salt and calorie intake restrictions and treatment with an angiotensin receptor blocker, multiple diuretics, and a sodium-glucose cotransporter-2 inhibitor, she experienced multiple hospitalizations due to congestive heart failure. Three months ago, her urine protein was elevated and her serum albumin concentration was decreased, resulting in the pathophysiology of nephrotic syndrome secondary to DKD. She took Saireito, a representative Kampo formula for nephrotic syndrome, for one month with no improvements.

**Figure 1:**
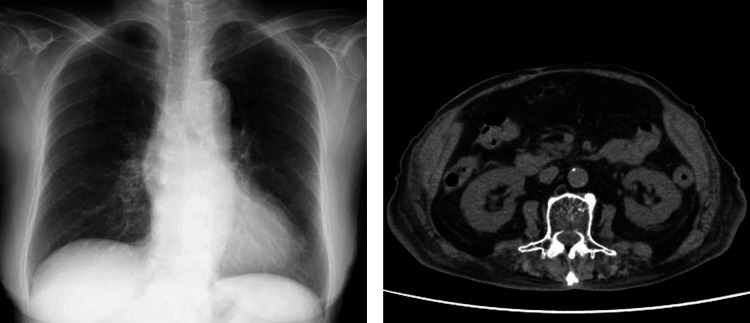
Chest X-ray and abdominal computed tomography before the initiation of shichimotsukokato

The patient’s height was 137.0 cm, and her weight was 44.1 kg, which was approximately 4 kg over her dry weight. Her blood pressure and pulse rate were 154/63 mmHg and 83 beats per minute, respectively. Physical examination revealed a pale complexion and weakness in the lower abdominal region. Although she had bilateral pitting edema in her lower extremities, her overall skin surface remained dry and exhibited a rough texture. Her daily medications comprised 100 mg of losartan, 80 mg of furosemide, 25 mg of spironolactone, 10 mg of empagliflozin, 5 mg of linagliptin, and 5 mg of rosuvastatin. Table 1 shows the results of baseline blood and urine tests, confirming nephrotic-range proteinuria and hypoalbuminemia. The estimated glomerular filtration rate, hemoglobin A1c concentration, and low-density lipoprotein cholesterol concentration were 20.7 mL/min/1.73 m², 7.1%, and 94 mg/dL, respectively. Chest X-ray showed that her cardiothoracic ratio was 51.3% (Figure 1). Computed tomography showed mild bilateral renal atrophy.

**Table 1:** Baseline blood and urine test results at the initiation of shichimotsukokato eGFR: estimated glomerular filtration rate; LPF: low power fields

Blood examination	Results	Reference range
White cell count, /µL	5700	3300-8600
Hemoglobin, g/dL	9.2	11.6-14.8
Hematocrit, %	27.6	35.1-44.4
Platelet, 10^4^/µL	27.8	15.8-34.8
Total protein, g/dL	5.6	6.6-8.1
Serum albumin, g/dL	2.1	4.1-5.1
Blood urea nitrogen, mg/dL	38.5	8.0-20.0
Serum creatinine, mg/dL	1.80	0.46-0.79
eGFR, mL/min/1.73m^2^	20.7	≥60
Uric acid, mg/dL	4.2	2.6-6.9
Aspartate aminotransferase, IU/L	17	13-30
Alanine aminotransferase, IU/L	8	7-23
Alkaline phosphatase, IU/L	58	124-222
Lactate dehydrogenase, IU/L	221	38-113
Triglycerides, mg/dL	205	30-149
High-density lipoprotein cholesterol, mg/dL	43.3	40.0-103.0
Low-density lipoprotein cholesterol, mg/dL	94	65-139
Hemoglobin A1c, %	7.1	4.7-6.1
C-reactive protein, mg/dL	0.02	<0.14
Urine examination	Results	Reference range
Urine protein/creatinine ratio, g/gCr	16.3	<0.15
Epithelial casts, per 100 LPF	1~	-
Granular casts, per 100 LPF	1~	-

**Figure 2:**
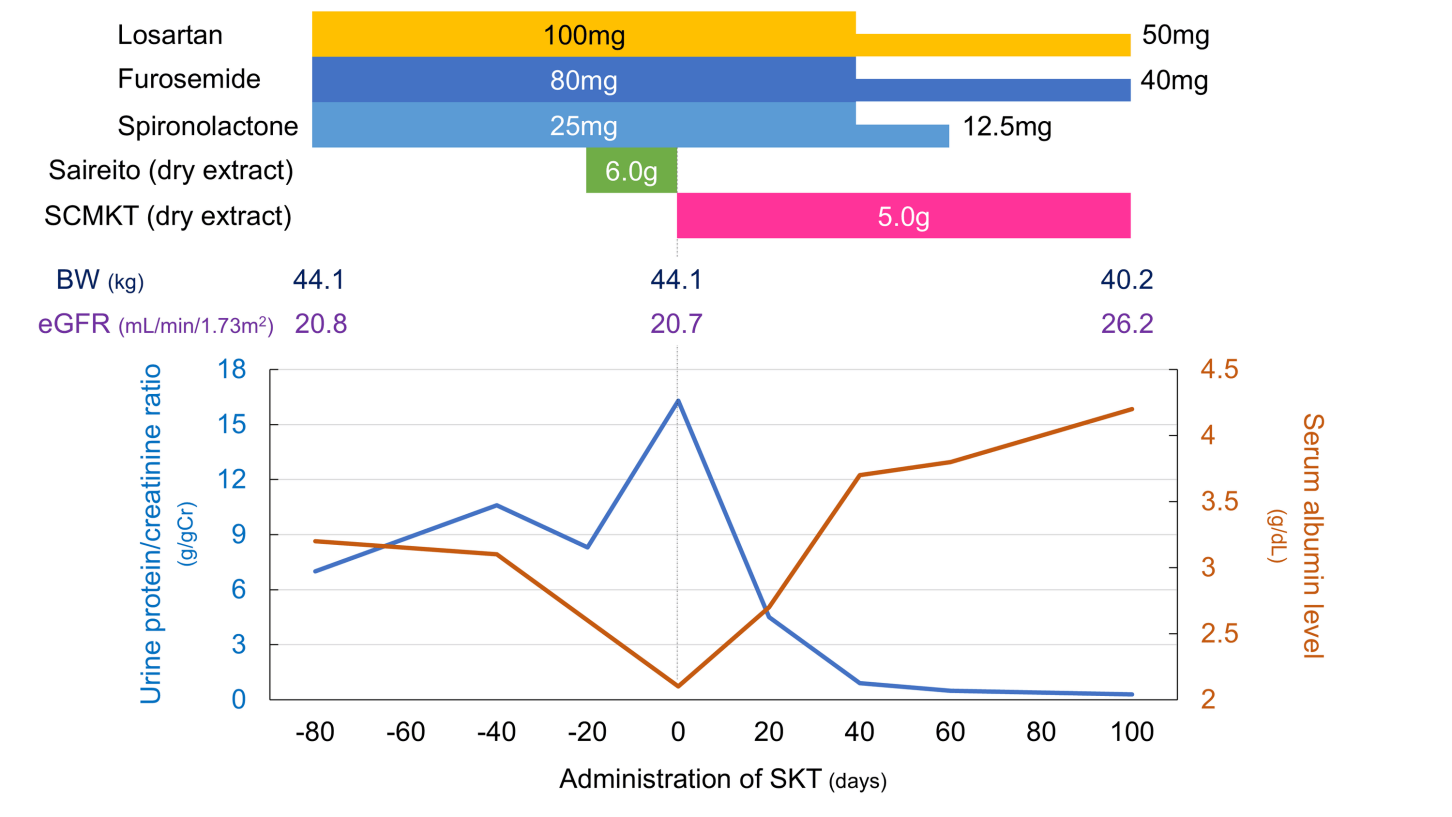
Clinical chart in this case Dosages of all medications are the daily dosages. SCMKT: shichimotsukokato; BW: body weight; eGFR: estimated glomerular filtration rate

On the basis of Kampo diagnostic principles, she was started on 5 g/day of an orally administered dry extract of SCMKT (Tsumura & Co., Tokyo, Japan), focusing on her age, pale complexion, dry skin, and hypertension. Following the initiation of SCMKT, her proteinuria rapidly decreased (Figure 2). Forty days after starting on SCMKT, her serum albumin concentration rose to 3 g/dL and the fluid overload improved without any other new drugs, allowing the tapering of losartan, furosemide, and spironolactone. Other administered drugs, including empagliflozin, linagliptin, and rosuvastatin, were continued during the SCMKT therapy. Table 2 shows the changes in the clinical indicators of nephrotic syndrome in this case. The amount of urinary protein decreased to the level of complete remission, and the serum albumin concentration recovered. Spironolactone was discontinued because the edema resolved. The estimated glomerular filtration rate improved from 20.7 to 26.2 mL/min/1.73 m². Moreover, her blood pressure was stable, and the dosage of losartan was reduced. One hundred days after the initiation of SCMKT, the patient was transferred to another medical facility because of the burden of long-distance hospital visits.

**Table 2:** Comparison of clinical indicators before and after shichimotsukokato administration eGFR: estimated glomerular filtration rate; LPF: low power fields

Values	Baseline	End of treatment
Body weight, kg	44.1	40.2
Blood pressure, mmHg	154/63	141/55
Serum albumin, g/dL	2.1	4.2
eGFR, mL/min/1.73m^2^	20.7	26.2
Triglycerides, mg/dL	205	282
Low-density lipoprotein cholesterol, mg/dL	94	76
Hemoglobin A1c, %	7.1	7.5
Urine protein/creatinine ratio, g/gCr	16.2	0.28
Epithelial casts, per 100 LPF	1~	-
Granular casts, per 100 LPF	1~	-

## Discussion

To the best of our knowledge, this is the first case reporting the effectiveness of SCMKT in treating nephrotic syndrome in a patient with DKD. The administration of SCMKT improved the patient’s proteinuria and hypoalbuminemia, resulting in the remission of nephrotic syndrome.

Crude drugs are natural substances derived from plants, animals, or minerals that have been processed explicitly for medical use in Kampo medicine. These materials are combined according to traditional concepts to enhance their therapeutic effects. SCMKT was created by the Japanese physician Keisetsu Otsuka to treat the pathophysiology of older hypertensive patients with blood deficiency patterns. The representative signs of blood deficiency patterns are pale complexion and dry skin, which were observed in this patient. SCMKT is composed of seven crude drugs (http://mpdb.nibiohn.go.jp/stork/). Among them, Astragalus root (*Astragali radix*) and Uncaria hook (*Uncariae Ramulus Cum Uncis*) are key drugs that treat the pathophysiology of DKD [10,11]. Previous studies have demonstrated that Astragalus root and its chief component, astragaloside IV, improve insulin resistance, renal pathological changes, and kidney function in a murine diabetic model [12-14]. Furthermore, clinical reports have suggested that Astragalus root has pharmacological benefits in treating nephrotic syndrome [15,16]. Uncaria hook and its components, such as hirsutine and rhynchophylline, also ameliorate insulin resistance and endothelial vascular dysfunction in animal models [17,18]. Although there is little evidence for the effectiveness of SCMKT in secondary nephrotic syndrome due to DKD, these crude drugs may have played a pivotal role in our case.

In contrast to SCMKT, Saireito did not work in our case. Saireito is traditionally used in Japan (http://mpdb.nibiohn.go.jp/stork/), and studies have indicated that Saireito is effective for chronic glomerular nephritis and primary nephrotic syndrome [7,19,20]. Saireito consists of 12 crude drugs but does not contain Astragalus root and Uncaria hook, which may have influenced the clinical outcome in this case.

Our report has several limitations. First, this was a single case report. Second, we did not obtain renal pathological data. Third, we did not evaluate the long-term effect of SCMKT on DKD. Fourth, our report did not provide the precise pharmacological mechanism of SCMKT. Fifth, we could not evaluate the interactions between SCMKT and other medications. Further clinical trials are needed to validate our findings and clarify the long-term efficacy and safety of SCMKT in treating nephrotic syndrome in patients with DKD.

## Conclusions

We reported a case in which SCMKT successfully achieved remission of secondary nephrotic syndrome in an older adult patient with DKD. SCMKT might be a valuable therapeutic option in similar cases. Further research is warranted to investigate the pharmacological mechanism of SCMKT on the pathophysiology of DKD.
